# The Impact of Academic Achievement and Parental Practices on Depressive Symptom Trajectories Among Chinese Adolescents

**DOI:** 10.1007/s10802-021-00826-9

**Published:** 2021-05-13

**Authors:** Xingna Qin, Tessa Kaufman, Lydia Laninga-Wijnen, Ping Ren, Yunyun Zhang, René Veenstra

**Affiliations:** 1 Collaborative Innovation Center of Assessment Toward Basic Education Quality, Beijing Normal University, Beijing, China; 2 Department of Sociology, University of Groningen, Groningen, The Netherlands; 3 Department of Pedagogy and Educational Sciences, Utrecht University, Utrecht, The Netherlands

**Keywords:** Depressive symptom trajectories, Chinese adolescents, Academic achievement, Parental autonomy support, Psychological control; educational involvement

## Abstract

Though depressive symptoms tend to increase in early adolescence, the trajectories of these symptoms may vary strongly. This longitudinal study investigated the extent to which the distinct developmental trajectories of depressive symptoms were predicted by adolescents' academic achievement and perceived parental practices in a sample of Chinese young adolescents (*N* = 2,576). The results showed four trajectory profiles of depressive symptoms: low-stable (75%), low-increasing (11%), high-stable (9%), and high-decreasing (5%). Adolescents with high academic achievement were more likely to be classified into the low-stable, low-increasing, and high-decreasing profiles than into the high-stable depressive symptom profile. Moreover, students who perceived greater parental *autonomy support* were more likely to be in the low-stable and low-increasing profiles than the high-stable profile, whereas adolescents perceiving more parental *psychological control* had higher odds of being in the low-increasing rather than the low-stable profile. Parental *educational involvement* was unrelated to students' depressive symptom trajectories. In sum, Chinese adolescents with higher academic achievement and who perceived more parental autonomy support, and less psychological control, were at lower risk of experiencing depressive symptoms.

**Supplementary Information:**

The online version contains supplementary material available at 10.1007/s10802-021-00826-9.

The prevalence of depressive symptoms increases from early adolescence onwards (Duchesne & Ratelle, 2014; Mezulis et al., 2014; Yaroslavsky et al., 2013). A growing body of evidence shows that adolescents vary strongly from each other in how their depressive symptoms develop. Most adolescents remain low in depressive symptoms, but some may experience an increase – either temporarily or persistently (Cumsille et al., 2015; Duchesne & Ratelle, 2014). Adolescents who experience an increase in depressive symptoms and – particularly – who are persistently high in depressive symptoms are at risk for several adverse outcomes, including comorbid mental health problems and physical complaints (see review, Musliner et al., 2016). Therefore, it is important to identify characteristics that predict which adolescents are most likely to experience such stable high or elevated depressive symptom trajectories.

In early adolescence, the development of depressive symptoms may relate to two critical developmental tasks: being granted autonomy from parents (Soenens & Vansteenkiste, 2010) and fulfilling one's academic potential (Crosnoe & Brenner, 2015). Adolescents start seeking autonomy from authority figures such as parents to establish their own identity (Veenstra & Laninga-Wijnen, 2021). Parents can support young adolescents by providing psychological support and granting age-appropriate autonomy (Ryan & Deci, 2017). However, when parents fail to do so, it may restrict adolescents' psychological needs for autonomy; this may result in distress and the development of psychosocial problems such as depressive symptoms (Chai et al., 2018). Moreover, early adolescence is accompanied by a transition to middle school, a type of education that is much more challenging academically compared with elementary school because of more homework, different teachers, and official tests (Evans et al., 2018). When students feel they are insufficiently able to meet these new demands (because they obtain low grades), this may trigger negative feelings about themselves and may result in increased depressive symptoms.

Indeed, various studies in Western countries found both low academic achievement (e.g., Huang, 2015) and parental control (e.g., Cheung & Pomerantz, 2011) to affect adolescents' depressive symptoms. Although these studies provide valuable insights into these processes in *Western countries,* little is known about the trajectories of depressive symptoms and the effects of academic achievement and parental practices on these trajectories in Eastern contexts, such as China. In China, academic achievement is viewed as a pathway to improving individuals' and their families' social status (Quach et al., 2013). Extremely high value is placed on academic achievement in China because opportunities for higher education depend primarily on adolescents' performance in exams (Cheung & Pomerantz, 2011). The weight that is put on achievement may have important implications for how it is related to youths' well-being and psychosocial functioning and, hence, depressive symptom trajectories. Moreover, parental practices, such as controlling or autonomy-restrictive practices, may be more normative in China than in Western contexts and, therefore, be viewed as appropriate by Chinese adolescents (Cheung & Pomerantz, 2011). Parental practices, such as increased involvement in children's learning and heightened control of their activities, are accepted as an act of love (Chao, 1994), and as such may not be as strongly related to depression in China as is the case in Western contexts. Therefore, our study aimed to clarify how Chinese adolescents' academic achievement and their perceptions of parental practices (autonomy support, psychological control, and educational involvement) shape trajectories of depressive symptoms after the transition to middle school.

## Depressive Symptom Trajectories during Adolescence

Researchers acknowledge that depressive symptoms develop heterogeneously across adolescence. Prior work has adopted a person-centered approach to analyze this heterogeneity of depressive symptom trajectories, and has focused almost exclusively on Western contexts (e.g., Musliner et al., 2016). Findings from studies examining depressive symptom trajectories in early adolescence vary somewhat due to the different samples and measures for assessing depressive symptoms. However, some general developmental tendencies have been observed. First, most studies distinguished three to four developmental trajectories that varied by severity (low, moderate, or high) or stability (stable, increasing, or decreasing) (Musliner et al., 2016). Second, the proportions of adolescents within these trajectories varied widely. The majority of adolescents (about 70%) had low or mild symptoms, a notable minority reported increasing or decreasing symptoms, and a small proportion (< 10%) experienced persistently high levels of symptoms over time (e.g., Duchesne & Ratelle, 2014; Mezulis et al., 2014; Reinke et al., 2012). Third, trajectories of depressive symptoms differed in the severity of risk to the individual's development. The low-stable trajectory is commonly regarded as a low-risk subgroup. However, the high-stable trajectory, rare but deleterious, is usually viewed as the most maladaptive subgroup because individuals with persistently high symptoms were more likely to be diagnosed with a depressive disorder (Musliner et al., 2016; Stoolmiller et al., 2005). Trajectories characterized by moderate, increasing or decreasing symptoms were also considered risk subgroups in prior work (though not as severe as the high-stable group), given their association with poor psychiatric and psychosocial outcomes in later life (Musliner et al., 2016; Yaroslavsky et al., 2013).

Only a few studies have focused on the trajectories of depressive symptoms among Chinese adolescents. These studies consisted mainly of respondents from upper socioeconomic regions, including Taiwanese samples (Wang et al., 2018; Wang et al., 2018; Wu, 2017). Their findings on trajectories of depressive symptoms are mostly consistent with Western findings. For example, Wu (2017) identified four distinct trajectories: stable-low, moderate, increasing, and early elevated but later decreasing symptoms among students from Grade 7 to Grade 9. Based on prior studies (e.g., Reinke et al., 2012; Wu, 2017), we expected to find similar developmental trajectories of depressive symptoms: that is, a low-risk trajectory (i.e., stable-low), moderate-risk trajectory (i.e., increasing, decreasing, or moderate), and high-risk trajectories (i.e., stable-high) during early adolescence in China. The main aim of this study was to examine this expectation among adolescents from a generally moderate socioeconomic background in Mainland China, in order to contribute to the information on the cross-country generalizability of previous findings.

## Academic Achievement and Depressive Symptoms

Low academic achievement is associated with subsequent high depressive symptoms (Huang, 2015; Stoolmiller et al., 2005); two theories could explain this. First, the stress process model (Pearlin, 1999) provides a theoretical framework to understand the psychological processes and negative influence of objective and subjective stressors. Based on this model, low academic achievement can be viewed as an objective stressor that enhances academic stress – a subjective stressor – thus resulting in an elevated depressive mood. A review found that academic achievement was negatively related to academic stress in the past 20 years in China (Ye et al., 2019), and that academic stress was positively associated with depressive symptoms among adolescents (Liu & Lu, 2012). Second, according to the competence-based model of depression (Cole, 1991; Vannucci & Ohannessian, 2018), adolescents' academic self-worth and self-evaluations have an impact on depressive symptoms. Low academic achievement is likely to be related to negative perceptions of competence, resulting in higher levels of depression. In contrast, high academic achievement can buffer against depressive symptoms because it boosts adolescents' academic self-competence.

To date, only a few studies have examined academic performance in relation to depressive symptom trajectories. One study found that adolescents (11–16 years of age) with high academic competence were more likely to be in a low-stable trajectory (Duchesne & Ratelle, 2014). Some researchers found, among a US sample aged from 16 to 24 (Stoolmiller et al., 2005), that youth with lower academic achievement in childhood were more likely to be in a persistently high symptom trajectory. Three studies found *no* significant effects of self-reported academic problems among a sample aged from 14 to 30 (Yaroslavsky et al., 2013), teacher-reported academic performance among students from Grade 2 to Grade 8 (Mazza et al., 2010), or academic competence during middle-to-late adolescence (Vannucci & Ohannessian, 2018). Altogether, there are tentative signals that lower academic competence is linked to adolescents' high depressive symptom trajectories.

It is particularly relevant to examine this among non-Western adolescents. In China, academic achievement is accepted as a means of self-cultivation and self-perfection, and an obligation to the family and even the whole society (Wang et al., 2018; Wang et al., 2018). Academic success is extremely important: those who excel academically have the chance to improve not only their own but also their family's reputation, whereas those who underperform might be considered failures and will face many difficulties in later life (Quach et al., 2013; Wang et al., 2018; Wang et al., 2018). Academic success thus brings protection, and academic underperformance puts pressure on Chinese adolescents. Therefore, examining the role of academic achievement in developmental trajectories of depressive symptoms in Chinese young adolescents provides an essential and novel addition to the literature.

## Parental Autonomy Support, Psychological Control, and Educational Involvement

Psychological control versus autonomy support is commonly used to describe parental practices (Chao, 1994; Cheung & McBride-Chang, 2008; Cheung & Pomerantz, 2011). Based on self-determination theory, autonomy is a fundamental need, and its satisfaction is essential to one's psychosocial adjustment (Ryan & Deci, 2002, 2017). Deficits in its fulfilment may relate to psychosocial maladjustment. From early adolescence onwards, young people start to seek autonomy, particularly in their relationships with their parents (Smetana & Daddis, 2002). Parents may vary, however, in the extent to which they grant autonomy to their children. Adolescents who experience high parental psychological control (e.g., having parents who withdraw love or induce guilt) may feel forced to behave, think, and feel in ways that fit their parents' desires. It may hinder them from fulfilling their need for autonomy, which may lead to emotional maladjustment (Soenens & Vansteenkiste, 2010), such as heightened depressive symptoms (Liu & Merritt, 2018). In contrast, higher levels of parental autonomy-granting may help adolescents in establishing their own identity, which may buffer them against the development of depressive symptoms. Indeed, a meta-analysis study has shown that parental autonomy support accounted for 16% of the variance in children's depression (Yap et al., 2014). However, the relations of parental psychological control and autonomy support with developmental trajectories of depressive symptoms have not been examined in early adolescence.

In addition, parental educational involvement, defined as parents' investment in children's academic lives, for example, discussing school-related topics with children and assisting with homework (Cheung & Pomerantz, 2011, 2015), may be related to the development of depressive symptoms during adolescence. When adolescents perceive educational involvement from their parents, they may feel supported by their parents and may become confident about themselves. Previous studies have indicated that parental educational involvement was positively associated with decreasing depressive symptoms among both US and Chinese adolescents (Cheung & Pomerantz, 2011).

## Cultural Differences

Parental practices (i.e., autonomy support, psychological control, and educational involvement) might have different meanings in Chinese culture than in other (Western) countries. Chinese parenting is typically characterized by a high level of parental control and centers on the child's academic achievement (Wang et al., 2018; Wang et al., 2018). First, profoundly affected by the Chinese parenting belief of "guan (管)", which involves training, discipline, supervision, governance, monitoring, and even control, Chinese parents express love and support by controlling children instead of through affection (Chao, 1994). Second, the importance of academic success is heavily emphasized by Chinese parents as a family obligation (Wang et al., 2018; Wang et al., 2018). Consequently, Chinese parents tend to focus their educational involvement, autonomy support, and psychological control on their children's learning achievements more than do their Western counterparts (Cheung & Pomerantz, 2011; Wang et al., 2007). They also devote substantial time and energy to children's learning (Li et al., 2020), such as checking children's homework against their will. Influenced by these cultural norms, Chinese adolescents may perceive parental control and involvement as an expression of love and care (Cheung & McBride-Chang, 2008; Cheung & Pomerantz, 2011).

In modern Chinese society, however, being the subject of high levels of parental psychological control is considered detrimental to children's and adolescents' psychological well-being and social-emotional development (Cheung & Pomerantz, 2011; Li et al., 2020). Moreover, given that adolescents strive for autonomy and independence (Smetana & Daddis, 2002), a lack of parental autonomy support may exacerbate adolescents' depressive symptoms (Liu & Merritt, 2018). In addition, several empirical studies have provided evidence for cultural similarities in China and the US in the association between parental practices and adolescents' development, such as well-being and academic functioning (Cheung & Pomerantz, 2011; Wang et al., 2007). It is thus worthwhile to examine whether and how parental practices (autonomy support, psychological control, and educational involvement) relate to Chinese adolescents' depressive symptom trajectories.

## The Current Study

Our study aimed to identify and predict the developmental trajectories of depressive symptoms among Chinese adolescents after the transition to middle school (grades 7 and 8). We expected to find at least three kinds of developmental trajectories: a low-risk trajectory (i.e., stable-low), a moderate-risk trajectory (i.e., increasing, decreasing, or moderate), and high-risk trajectories (i.e., stable-high). Next, we focused on the extent to which depressive symptom trajectories were predicted by adolescents' achievement, parental psychological control and autonomy support, and educational involvement. We hypothesized that high academic achievement would predict trajectories of low-risk depressive symptoms. Based on self-determination theory, the Chinese national tradition of guan, and the previous findings (Cheung & Pomerantz, 2011), we hypothesized that high parental autonomy support and educational involvement would be associated with the low-risk trajectories, while high psychological control would be associated with the high-risk depressive symptom trajectories.

In addition, we considered various potentially confounding factors: personal (gender and age), socioeconomic (parents' highest level of education and subjective family financial status), and school characteristics (rural/urban school). Moreover, in additional analyses, we considered interaction effects with gender. Girls are often more likely than boys to develop depressive symptoms (Musliner et al., 2016), and previous researchers found that Chinese girls were more distressed academically than boys (Liu & Lu, 2012), whereas Chinese boys were more distressed by over-controlling parents (Shek, 2008). Furthermore, differences between urban and rural schools may be related to depressive symptoms in Chinese adolescents, in the sense that a higher prevalence of depressive symptoms may be found in rural areas because of poor social support and poor living conditions (Rao et al., 2019).

## Method

### Participants and Procedure

Participants were recruited from seven randomly selected public general middle schools located in Central China. In total, 13 classrooms in two urban schools, 15 classrooms in one suburban school, and 19 classrooms in four rural schools were randomly selected. Four waves of data were collected at six-month intervals, starting in January 2015 at the end of the first semester in Grade 7, the first year of middle school. The initial sample was 2,613 at T1. In total, 35 students (1%) did not complete their survey, and two students had reading problems, who were excluded from further analysis. Those who did not participate at T1 (*n* = 222) were also excluded because the predictors were at T1. Thus, a total of 2,576 students (53% boys) were included in the final analysis, with a mean age of 13.0 (*SD* = 0.6) at T1. Around 2,544 students participated at T2, 2,468 at T3, and 2,389 at T4, due to participants' dropping out or moving to another school.

Parents or legal caregivers were informed about this research and were asked to provide informed consent for their child’s participation in the research. Students’ informed assent was required and they participated voluntarily. Students did not participate when they had no permission from their parents or caregivers, when they did not want to participate themselves, or when they were unable to complete the questionnaire. Students filled in the questionnaire in their classrooms and were instructed by trained postgraduate or undergraduate students. The classroom supervisor was also present and responsible for the students' discipline while doing the survey.

## Measures

**Depressive Symptoms.** were measured using a Chinese version of the Child Depression Inventory (CDI; Kovacs, 1992). The CDI is a self-rated 27-item scale for children aged 7–17, which assesses feelings or behaviors associated with depressive symptoms in the previous two weeks: for example, loneliness, self-blame, sleep disturbance, and reduced appetite. The items scored from 0 to 2. We used the mean score, with a higher score indicating a higher level of depressive symptoms. The cut-off point for the screening of depression is 0.70 (which reflects a score of 19 or higher on the 27 items). The CDI has proven reliable and valid among Chinese adolescents (Rao et al., 2019). Although no nationally representative norms exist, the cut-off point of 19 is widely used and usually considered as an indicator of high depressive symptoms (or high risk of depression) in China (Wang et al., 2016). The Cronbach's alpha ranged from 0.87 (T1) to 0.89 (T4). Furthermore, the results of longitudinal measurement invariance of CDI supported residual invariance (See Appendix).

**Academic Achievement.** was obtained from the school records and included seven main subjects: Chinese, Mathematics, English, history, geography, biology, and politics. The scores were derived from the objective examinations conducted at the end of the first semester in Grade 7. The scores were first standardized per subject and then overall averaged to form an index of academic achievement. The reliability (Cronbach's alpha) of the seven subjects was 0.91 at T1.

**Parental Psychological Control.** was assessed using 18 items about perceived parental control (Wang et al., 2007) at T1. Ten items assessed guilt induction (e.g., "My parents tell me that I should feel guilty when I do not meet their expectations."). Five items were for love withdrawal (e.g., "My parents act cold and unfriendly if I do something they do not like."). Three items were for authority assertion (e.g., "My parents tell me that what they want me to do is the best for me and I should not question it."). Students indicated (0 = *not at all*; 4 = *very often*) how often their parents used these psychological control practices. All items were averaged, with higher scores reflecting greater perceived psychological control (α = 0.90).

**Parental Psychological Autonomy Support.** was assessed using eight items about parental psychological autonomy-supportive behaviors (Wang et al., 2007) at T1. Four items tapped choice-making (e.g., "My parents allow me to make choices whenever possible.") and four tapped opinion exchange (e.g., "My parents encourage me to give my ideas and opinions when it comes to decisions about me."). Students responded to each item by indicating how often their parents endorsed autonomy support (0 = *not at all*; 4 = *very often*). We took the mean of these items, with higher scores reflecting more perceived autonomy support from their parents (α = 0.87).

**Parental Educational Involvement.** was assessed using ten items about parents' involvement in children's learning (Cheung & Pomerantz, 2011) at T1. A sample item is, "My parents try to get to know the teachers at my school." Students reported for each item how often their parents showed the described behaviors (0 = *not at all*; 4 = *very often*). We used the average of these items, with higher scores indicating greater perceived parental involvement in students' learning (α = 0.84).

**Parents' Highest Level of Education.** was reported by students at T1, for fathers and mothers separately, with eight levels from "never been in school" to "master's degree or higher." The education level was computed as the number of years it takes to complete that level. In the end, we took the highest education level of either the father or the mother as the parents' highest level of education. Adolescent-reported parental education is widely used in various studies, including the American National Study of Learning Mindsets (NSLM; Yeager et al., 2019) and in Chinese research (Long & Pang, 2016).

**Subjective Socioeconomic Status (SES).** was measured on a five-point scale using the question: "How would you rank your family's financial situation?" at T1. Responses were rated from 1 to 5, representing low, middle-low, middle, middle-high, and high. A meta-analysis has shown that subjective SES is an indicator of family wealth and is associated with adolescents' health outcomes (Quon & McGrath, 2014).

Rural, urban, and suburban schools were defined according to the locations in Central China; two dummy variables were created, one for urban and one for rural schools, with the suburban school as the reference category. There were 927 students in rural schools, 739 in urban schools, and 910 in suburban schools.

## Strategy of Analysis

Analyses were conducted using M*plus* version 7.11 (Muthén & Muthén, 2015). Growth Mixture Modelling (GMM) was used to identify the trajectories of depressive symptoms. Through GMM, it is possible to identify different trajectory profiles that may change over time. We started with a one profile model and increased the number of profiles until a parsimonious model with a good model fit was reached. Several model fit criteria were considered when selecting the best-fitting number of profiles of depressive symptoms. Beysain Information Criterion (BIC), Akaikes Information Criteria (AIC), and sample size adjusted BIC (Adj.BIC) were examined to evaluate relative model fit, with lower values indicating better model fit. Lo-Mendell-Rubin Adjusted Likelihood Ratio (LMR-LRT), Vuong-Lo-Mendell-Rubin Likelihood Ratio (VLMR), and the bootstrapped likelihood ratio test (BLRT) were used to compare the estimated *k* class model with the *k* – 1 class model. A significant *p*-value indicated that the estimated *k* class model was superior. Entropy measured classification accuracy, with values closer to 1 indicating better accuracy.

Having selected the best fitting model, we used a three-step GMM procedure (R3STEP), called Mplus AUXILIARY, to examine the effects of individual and parental predictors on different trajectories of depressive symptoms. The three-step GMM procedure considers the uncertainty associated with group membership when this is used as the dependent variable (Asparouhov & Muth, 2018). It adds the predictors (covariates) of group membership simultaneously, which also incorporates within-group variation as a random effect (Muthén, 2004). In this multinomial logistic regression model, we examined the role of academic achievement and parental practices (psychological control, autonomy support, and educational involvement) in depressive symptom trajectories, taking gender, age, parents' highest education, subjective SES, and urban/rural schools into account. In additional analyses, we examined the interactive effects of academic achievement and parenting practices with the gender on depressive symptom trajectories. None of these interaction effects was significant; they were, therefore, erased from the models. The findings of these additional analyses are available on request from the first author. The False Discovery Rate method (FDR Benjamini & Hochberg, 1995) was used to account for multiple comparisons in the three-step GMM procedure.

## Missing Data

The percentages of participants with missing data were 9%, 2%, 6%, and 9% across the four waves, respectively. We compared participants with and without missing values. Students with missing values had lower academic achievement (*t*(2536) = -13.30, *p* < 0.001, Cohen’s *d* = 0.75), perceived less parental autonomy support (*t*(2543) = -5.32, *p* < 0.001, Cohen’s *d* = 0.34), less educational involvement (*t*(2543) = -4.35, *p* < 0.001, Cohen’s *d* = 0.29), more psychological control (*t*(2543) = 2.66, *p* = 0.008, Cohen’s *d* = 0.18), and showed more depressive symptoms at T1 (*t*(2542) = 5.30, *p* < 0.001, Cohen’s *d* = 0.29), at T2 (*t*(2481) = 4.42, *p* < 0.001, Cohen’s *d* = 0.28) and T3 (*t*(2396) = 2.96, *p* = 0.003, Cohen’s *d* = 0.25) than those without missing values. However, students with and without missing values did not differ in terms of depressive symptoms at T4 (*t*(2328) = 0.75, *p* = 0.45, Cohen’s *d* = 0.16).

In all models, we used maximum likelihood estimation (MLR) with robust standard errors, which is robust to violations of normality. Full Information Maximum Likelihood estimation (FIML) was only used for students with missing values in depressive symptoms. FIML cannot be applied to missing data in predictor variables because the AUXILIARY approach estimates the GMM model and prediction model separately (Asparouhov & Muth, 2018). Therefore, participants with missing data on the predictors at T1 were excluded using the listwise deletion AUXILIARY approach in Mplus.

## Results

### Descriptive Results

Descriptive statistics and correlations of all variables appear in Table 1. The mean score of depressive symptoms ranges from 0.49 to 0.53 across time. As for correlations, depressive symptoms correlated weakly negatively with academic achievement (*r*s varied from -0.13 to -0.17, *p* < 0.001), moderately negatively with parental autonomy support (rs varied from -0.21 to -0.33, *p* < 0.001), weakly to moderately negatively with educational involvement (*r*s varied from -0.16 to -0.26, *p* < 0.001), and weakly positively with psychological control (*r*s varied from 0.14 to 0.19, *p* < 0.001). In terms of parents' highest level of education and subjective SES, 73% of the participants' fathers and 75% of their mothers had a middle-school education level at the most. Most students (90%) ranked their SES as moderate at the most.

**Table 1:** Descriptive and Correlations among All Variables

Variables	M (SD)	1	2	3	4	5	6	7	8	9	10	11
1. Depressive symptoms T1	0.49 (0.29)											
2. Depressive symptoms T2	0.53 (0.31)	0.64^**^										
3. Depressive symptoms T3	0.52 (0.31)	0.56^**^	0.68^**^									
4. Depressive symptoms T4	0.52 (0.32)	0.51^**^	0.62^**^	0.69^**^								
5. Academic achievement T1	-0.02 (0.83)	-0.17^**^	-0.14^**^	-0.14^**^	-0.13^**^							
6. Parental autonomy support T1	3.52 (0.87)	-0.33^**^	-0.27^**^	-0.25^**^	-0.21^**^	0.19^**^						
7. Parental psychological control T1	2.76 (0.77)	0.19^**^	0.18^**^	0.14^**^	0.15^**^	-0.17^**^	-0.24^**^					
8. Parental educational involvement T1	3.39 (0.76)	-0.26^**^	-0.20^**^	-0.17^**^	-0.16^**^	0.15^**^	0.50^**^	-0.00				
9. Parents’ highest education T1	10.10 (2.30)	-0.05^*^	-0.01	-0.01	-0.03	0.13^**^	0.13^**^	-0.04	0.18^**^			
10. Subjective SES T1	3.27 (0.74)	0.07^**^	0.06^**^	0.05^*^	0.05^*0^	.09^**^	-0.09^**^	-0.00	-0.06^**^	-0.17^**^		
11. Age T1	13.0 (0.62)	0.04^*^	0.05^*^	0.04	0.02	-0.19^**^	-0.07^**^	0.05^**^	-0.13^**^	-0.16^**^	-0.02	
12. Gender	0.48 (0.50)	0.00	0.02	0.05^**^	0.05^*^	0.19^**^	0.05^*^	-0.11^**^	0.01	-0.01	0.01	-0.08^**^

Parents' highest education level was transferred as the number of years that it takes. Compulsory education is usually nine years (six years of primary and three years of middle school education) in China

*two-tailed *p*<0.05; **p< 0.01; ***p<0.001

## Trajectories of Depressive Symptoms

We estimated fit indices for the depressive symptoms trajectory models (Table 2) using the three-step approach. From the two- to the four-profile model, each model improved in terms of decreasing AIC, BIC, and *Adj.*BIC values and significant BLRT, LMR, and VLMR values. The LMR and VLMR were not significant for the five-profile model, indicating that a five-profile model was not an improvement over the four-profile model. The entropy for the four-profile solution was 0.74, which was an acceptable level of classification accuracy (Vannucci & Ohannessian, 2018). Furthermore, the four-profile linear trajectory model aligns with previous research on depressive symptom trajectories and theories. Therefore, the four-profile solution was chosen for further investigation.

**Table 2:** Model Fit Statistics for All Growth Mixture Models with Admissible Solutions

								Profile Size (Min–Max)	
Solution	AIC	BIC	*Adj*.BIC	LMR	VLMR	Entropy	BLRT	Min	Max
1 profile	-102.10	-55.27	-80.68						
2 profile	-313.23	-248.84	-283.79	< .001	< .001	.73	< .001	384 (15%)	2144 (83%)
3 profile	-453.89	-371.93	-416.41	< .001	< .001	.71	< .001	276 (10%)	1980 (77%)
**4 profile**	**-516.66**	**-417.14**	**-471.16**	** < .001**	** < .001**	**.74**	** < .001**	**126 (5%)**	**1937 (75%)**
5 profile	-546.53	-429.45	-492.99	.06	.05	.74	< .001	35 (1%)	1798 (70%)

The best-fitting profile solution is in bold

*BIC*  Bayesian Information Criterion *LMR*  Lo-Mendelll-Rubin Adjusted Likelihood Ratio Test *VLMR*  Vuong-Lo-Mendell-Rubin Likelihood Ratio Test *BLRT*  the bootstrapped likelihood ratio test Entropy values *LMR* and *VLMR* are not available for a one-profile solution

**Fig. 1:**
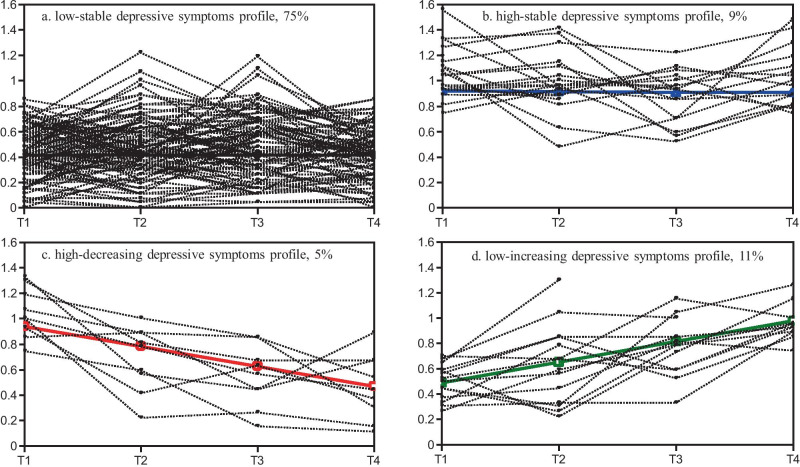
Trajectories of Depressive Symptoms Across Four Time Points with Estimated Percentages and Observed Individual Values (the number of random subsamples is 150). A, b, c, and d represents low-stable, high-stable, higi-increasing and low-increasing depressive symptoms profile seperately. The solid lines with different colurs present the estimated means and the dash lines for observed individual values in different profiles

As shown in Table 3, most students (*N* = 1,937, 75%) were identified as belonging in the first profile, labelled as low-stable with a low initial level of depressive symptoms (*b*_inter._ = 0.41, *p* < 0.001), which remained low through all assessments; this can be considered a "low-risk profile". The second profile was the high-stable profile (*N* = 234, 9%), characterized by a high initial level of depressive symptoms (*b*_inter._ = 0.92, *p* < 0.001) which maintained over time. The estimated means for the four assessments in this trajectory were around 0.9. Based on the CDI clinical cut-off value of 0.7 (Kovacs, 1992), this can be interpreted as "high depressive symptoms". Accordingly, this profile is considered "high-risk". The third profile was a low-increasing profile (*N* = 279, 11%), with low CDI cut-off scores at Grade 7 (*b*_inter._ = 0.49, *p* < 0.001), which increased in the subsequent year (*b*_slope_ = 0.16, *p* < 0.001) and were higher than the CDI clinical cut-off value in the end. The high-decreasing profile (*N* = 126, 5%) was characterized by high initial depressive symptoms (*b*_inter._ = 0.94, *p* < 0.001) which decreased over time (*b*_slope_ = -0.16, *p* < 0.001); see also Fig. 1. Compared with the low-risk profile, the two unstable profiles (low-increasing and high-decreasing) were considered "moderate-risk profiles", because the adolescents in these two profiles suffered high depressive symptoms (above the cut-off value) at certain time points, but not consistently at all moments.

**Table 3:** Adjusted Estimates of the Intercept and Slope Factors in the Depressive Symptom Growth Mixture Modeling

Profiles	Intercept				Slope		
	*b*	95% *CI*	*P*		*b*	95% *CI*	*p*
Profile 1 (low-stable depressive symptoms, *N* = 1937)	0.41	0.39–0.43	< 0.001		0.00	-0.01–0.01	0.99
Profile 2 (high-stable depressive symptoms, *N* = 234)	0.92	0.85–0.99	< 0.001		-0.01	-0.44–0.03	0.75
Profile 3 (low-increasing depressive symptoms, *N* = 279)	0.49	0.44–0.53	< 0.001		0.16	0.14–0.19	< 0.001
Profile 4 (high-decreasing depressive symptoms, *N* = 126)	0.94	0.87–0.10	< 0.001		-0.16	-0.20- -0.12	< 0.001

## Predictors of Depressive Symptom Trajectories

As hypothesized (see Table 4), students with high academic scores were significantly more likely to be categorized into the low-stable (*OR* = 1.93, *95% CI* [1.32, 2.82], *p* = 0.001) than the high-stable depressive symptom trajectory. Furthermore, high-achieving students were significantly more likely to be classified into low-increasing (OR = 1.85, *95% CI* [1.12, 3.04], *p* = 0.016) and high-decreasing profiles (OR = 2.72, *95% CI* [1.29, 5.71], *p* = 0.008) than into the high-stable depressive symptom profile. In other words, low-achieving adolescents had higher odds of being in the high-risk depressive symptom profile relative to the moderate-risk profiles.

**Table 4:** Predictions of Depressive Symptom Trajectories among Chinese Adolescents

Predictors in T1	low-stable Vs high-stable				low-increasing Vs high-stable				high-decreasing Vs high-stable				low-increasing Vs low-stable				high-decreasing Vs low-stable				low-increasing Vs high-decreasing		
	*OR*	95% *CI*	*Adj.p*		*OR*	95% *CI*	*Adj.p*		*OR*	95% *CI*	*Adj.p*		*OR*	95% *CI*	*Adj.p*		*OR*	95% *CI*	*Adj.p*		*OR*	95% *CI*	*Adj.p*
Academic achievement	**1.93**	**1.32–2.82**	**0.006**		**1.85**	**1.12–3.04**	**0.03**		**2.72**	**1.29–5.71**	**0.02**		0.96	0.73–1.27	0.78		1.41	0.81–2.45	0.26		0.68	0.38–2.56	0.26
Parental autonomy support	**2.23**	**1.48–3.35**	** < 0.001**		**1.87**	**1.13–3.09**	**0.04**		1.47	0.67–3.23	0.39		0.84	0.64–1.10	0.32		0.66	0.38–1.14	0.27		1.27	0.73–1.20	0.39
Parental psychological control	0.65	0.43–1.00	0.14		0.92	0.55–1.53	0.94		0.90	0.36–2.24	0.94		**1.41**	**1.11–1.79**	**0.03**		1.37	0.72–2.59	0.67		1.03	0.55–2.21	0.94
Parental educational involvement	1.27	0.81–0.99	0.51		1.32	0.75–2.34	0.51		0.89	0.39–2.05	0.80		1.04	0.77–1.40	0.80		0.70	0.40–1.22	0.51		1.48	0.84–1.92	0.51
Covariates																							
Gender (0 = male)	0.55	0.32–0.95	0.20		0.67	0.34–1.32	0.47		0.64	0.27–1.53	0.47		1.22	0.83–1.78	0.47		1.16	0.64–2.09	0.75		1.05	0.55–2.62	0.88
Age	0.96	0.61–1.51	0.85		0.90	0.49–1.64	0.85		1.12	0.48–2.62	0.85		0.94	0.65–1.35	0.85		1.17	0.65–2.09	0.85		0.80	0.43–1.99	0.85
Parents’ highest education	0.95	0.83–1.09	0.69		1.00	0.85–1.18	0.98		0.91	0.71–1.16	0.69		1.06	0.97–1.15	0.69		0.96	0.82–1.12	0.72		1.10	0.94–1.48	0.69
Subject SES	0.87	0.63–1.21	0.83		1.12	0.76–1.67	0.84		1.08	0.63–1.83	0.84		1.28	1.00–1.65	0.29		1.23	0.85–1.79	0.83		1.04	0.69–1.58	0.84
School location (rural)	1.74	0.87–3.48	0.24		2.57	1.07–6.17	0.21		1.76	0.50–6.20	0.48		1.48	0.91–2.39	0.24		1.01	0.43–2.36	0.99		1.47	0.60–3.57	0.48
School location (urban)	1.63	0.61–4.39	0.61		2.07	0.61–7.02	0.61		2.14	0.38–9.06	0.61		1.27	0.72–2.23	0.61		1.31	0.46–3.72	0.73		0.97	0.33–2.80	0.85

Comparison profile is the group after "Vs" for all analysis. The locations of the schools (rural/urban) are two dummy variables with suburban as the reference category. *OR* is for the log odds ratio, which is used as an index of effect size. Degrees of freedom for each comparison were 2330. Bold statistics are the significant effects. *Adj.p* is adjusted for multiple comparisons using the False Discovery Rate (*FDR*)

Regarding parental factors, mostly in line with our hypotheses, adolescents who perceived greater parental autonomy support had significantly higher odds of being in the low-stable (*OR* = 2.23, *95% CI* [1.48, 3.35], *p* < 0.001) and low-increasing (*OR* = 1.87, *95% CI* [1.13, 3.09], *p* = 0.015) profiles relative to the high-stable profile. Students perceiving more parental psychological control had greater odds (*OR* = 1.41, *95% CI* [1.11, 1.79], *p* = 0.005) of being in the low-increasing profile relative to the low-stable profile. Parental educational involvement was not associated with depressive symptom trajectories (*ps* > 0.05).

The control variables (gender, age, parents' highest level of education, subjective SES, and urban/rural schools) were unrelated to adolescents' depressive symptom trajectories (*ps* > 0.05). In addition, neither academic achievement (*ps* > 0.05) nor parental practices (*ps* > 0.05) differentiated between the low-stable and high-decreasing profiles or the low-increasing and high-decreasing profiles.

## Discussion

This study aimed to identify developmental trajectories of depressive symptoms and to examine whether academic achievement and parental practices (i.e., autonomy support, psychological control, and educational involvement) contributed to the likelihood of adolescents in Chinese middle school being in a certain developmental trajectory. As hypothesized, the results revealed four trajectories of depressive symptoms, with low-stable, low-increasing, high-stable, and high-decreasing profiles. Adolescents with high academic achievement and high autonomy support from their parents were less likely to be in the high-stable profile. Adolescents perceiving more parental psychological control had higher odds of being in the low-increasing profile relative to the low-stable profile. Parents' educational involvement was unrelated to adolescents' trajectories of depressive symptoms. Our results reveal the heterogeneity of depressive symptom development among Chinese young adolescents and highlight the differential roles of academic achievement and parental practices in distinguishing these heterogeneous trajectories of depressive symptoms.

## Developmental Trajectories of Depressive Symptoms in Early Adolescence

Consistent with the findings of most previous studies of adolescence (e.g., Cumsille et al., 2015; Reinke et al., 2012), the majority of adolescents (about 75%) were classified into the low-stable profile, which is the low-risk profile due to consistently low levels of depressive symptoms (below the CDI cut-off value; Kovacs, 1992). However, approximately 9% of adolescents experienced persistently high depressive symptoms (above the cut-off value) in Grade 7 and Grade 8. Furthermore, a considerable number of adolescents suffered unstable depressive symptoms (in moderate-risk profiles), either high (above the cut-off value) at the start but decreasing over time (around 5%), or low in the beginning but increasing and above the cut-off value in Grade 8 (about 11%). The percentages of the high-risk profile and moderate-risk profiles are consistent with those found in a previous study, which showed that the prevalence of depression was 24% based on CDI in a sample of 7–17-year-old children and adolescents in west-central China (Wang et al., 2016). Furthermore, our study adds to these findings that varying trajectories of depressive symptoms exist, and future studies are encouraged to examine later psychosocial and psychiatric outcomes associated with these trajectories. In particular, it would be interesting to examine whether the high-decreasing profile might be *less* risky compared with the low-increasing profile, given that the decreasing symptoms may signal that the adolescents are recovering from depressive symptoms and that they may have developed coping skills to handle negative events or emotions.

## Predictors of Developmental Trajectories of Depressive Symptoms

Consistent with our expectation that performing well academically would be related to low-risk profiles, we found that students with high academic achievement had higher odds of belonging to the low-risk profile (low-stable) relative to the high-stable profile. This finding is consistent with those of a US study among 15-to-24-year-olds that found that participants who had had lower academic achievement in childhood were more likely to be in persistently high depressive symptom trajectories (Stoolmiller et al., 2005). These comparable findings in both the Chinese and the US samples may demonstrate the generalizable importance of academic achievement in the development of depressive symptoms. Furthermore, it is noteworthy that high-achieving adolescents were also more likely to be in the low-increasing and high-decreasing profiles (the moderate-risk trajectories) than in the high-risk trajectory (high-stable). It is possible that high academic achievement comes at a cost for some adolescents. The cost for the high-achieving students may be more academic burdens, such as more homework, more examinations and high expectations from parents. These academic burdens may increase academic pressure (Sun et al., 2013), which may, in turn, elicit feelings of distress and internalizing problems such as depressive symptoms. Nevertheless, high academic achievement seemed to buffer against being in the high-risk trajectory (high-stable), indicating that academic achievement mostly appears to be a protective factor.

Regarding parental practices, high perceived parental autonomy support increased the odds of belonging in the low-stable profile rather than the high-stable depressive symptom profile. This finding is in line with our hypothesis that parental autonomy support would be associated with membership in the low-risk trajectory. It is consistent with self-determination theory, which identifies autonomy support as a parental practice that promotes youth's optimal social-emotional functioning (Ryan & Deci, 2002). We also found that adolescents who perceived high levels of parental autonomy support had higher odds of belonging to the low-increasing profile compared with the high-stable profile. Even though the low-increasing profile might be considered less maladaptive than the high-stable profile, this finding may indicate that, for some adolescents, higher levels of autonomy-granting work out adversely to some extent. A potential explanation is that these adolescents feel that their parents give them *too much* autonomy or that their parents expect them to independently take decisions about issues that are not age-appropriate (Li et al., 2020; Ryan & Deci, 2017). They may feel alone in coping with these decisions or tasks, which may trigger depressive symptoms over time. Future studies are encouraged to examine whether receiving too-high levels of autonomy support from parents increases adolescents' feelings that they are alone in making important decisions, which in turn may elicit depressive symptoms.

Consistent with our hypothesis, adolescents who perceived high levels of parental psychological control were more likely to experience depressive symptoms over time, even if they had a low level of symptoms in the beginning. Psychological control may hinder adolescents in the establishment of independence and autonomy, which become fundamental developmental needs from early adolescence onwards (Smetana & Daddis, 2002). Not being able to fulfil this need may negatively affect adolescents' psychosocial adjustment, as indicated by the increase of depressive symptoms in our study. Although prior work suggested that Chinese children may be bothered *less* by parents' controlling behavior given that it is more accepted and normative within their culture (Cheung & McBride-Chang, 2008). Our findings indicate that even among Chinese adolescents, parental psychological control may have adverse consequences. It thus seems that high parental psychological control is a common risk factor for the development of depressive symptoms as adolescents mature.

Contrary to our hypothesis, we found no effect of parental educational involvement on adolescents' trajectories of depressive symptoms. A potential explanation for this non-significant finding may be that the items used in our study reflect parents' investment in children's learning, which may measure the *quantity* rather than the *quality* of educational involvement (Cheung & Pomerantz, 2015). Parents can be highly involved in their adolescents' academics: for example, buying learning materials for their children (i.e., high quantity); however, some such parents can also be relatively critical or demanding, possibly increasing adolescents' stress and hence their depressive symptoms (Li et al., 2020). Other parents who show high levels of educational involvement may provide high-quality feedback or positively stimulate their children and affirm their academic competence. Therefore, future work may profit from capturing the *quality* of parental educational involvement.

As for the effects of covariates, no significant effects of control variables in individual, family, and school characteristics were found in this study. Perhaps gender only predicts depressive symptom trajectories at higher ages, as previous studies have found that the most significant increase in gender differences occurs between the ages of 15 and 18 years (Essau et al., 2000). Further, the non-significant effects of parents' highest education, subjective SES, and urban/rural schools may be related to our relatively homogeneous sample, which may have prevented us from detecting significant effects. This sample was from Central China and had a moderate socioeconomic status, with most parents having a low level of education.

## Strengths, Limitations, and Suggestions for Future Research

Our study has several strengths. The first strength is that we examined trajectories of depressive symptoms and their predictors in a relatively understudied population: Chinese young adolescents. Our findings mostly align with those of prior studies conducted in Western contexts, indicating commonalities in these processes despite potential cultural differences. Another strength is that the investigated predictors of depressive symptoms trajectories point to two critical developmental tasks in early adolescence: establishing autonomy from parents and doing well academically. Our findings indicate that not being able to fulfil these developmental tasks poses a risk for adolescents.

The practical implications of our findings emphasize the importance of identifying at-risk adolescents to improve prevention and intervention efforts. Prevention and interventions targeting adolescents who are at high risk for the development of depressive symptoms are of the utmost importance for the mental health of both the adolescents themselves and their families. Programs that target depressive symptoms could also consider achievement and parental practices as indicators: for example, through assessing academic results or parenting pre-and post-intervention to examine the potential factors that may need to change. For instance, if it becomes clear that young people with persistently high levels of depressive symptoms experience high levels of parental psychological control, not only the adolescents but also their parents should receive assistance.

This study has several limitations, and we wish to provide suggestions for future studies. First, the predictors were measured only at the first wave and were considered as time-invariant variables, leading to a limited understanding of the dynamic changes of these characteristics in the course of the development of depressive symptoms. Second, the short age span of this study (13–14 years) covers only a part of adolescence. Further research would benefit from expanding the age range from early to late adolescence, particularly for adolescents in the East Asian context whose academic pressure increases over time due to the college entrance examination. Third, the sample consisted of adolescents from a moderately developed area in China, and most participants' parents had received little education, which narrows the generalizability of the results to other (Chinese) populations or other Asian countries. Lastly, caution is necessary when interpreting the results given the relatively small associations of academic achievement and parental practices with depressive symptoms. These results were, however, in line with those of a meta-analysis that found a small association between academic achievement and depression (Huang, 2015), as well as small effects of parental practices on depressive symptoms (Liu & Merritt, 2018). It should be noted that other academic characteristics, such as perceived achievement, academic pressure, and academic competence, and parental characteristics, such as parental academic pressure, may also be associated with adolescents' depressive symptom trajectories.

In conclusion, our findings reveal the presence of four distinct trajectories of depressive symptoms among Chinese young adolescents: high-stable, low-increasing, low-stable, and high-decreasing profiles. Moreover, our study highlights that academic achievement, parental autonomy support, and psychological control are related to change and stability in depressive symptoms during early adolescence. Specifically, academic success in school, greater parental autonomy support, and less psychological control are associated with fewer depressive symptoms among adolescents.

## Supplementary Information

Below is the link to the electronic supplementary material.

Supplementary file1 (DOCX 20 KB)

Supplementary file2 (CSV 43 KB)

Supplementary file3 (OUT 405 KB)

Supplementary file4 (OUT 18 KB)

Supplementary file5 (OUT 25 KB)

Supplementary file6 (OUT 29 KB)

Supplementary file7 (OUT 34 KB)

Supplementary file8 (OUT 38 KB)
