# Supplementary material for: The Impact of Academic Achievement and Parental Practices on Depressive Symptom Trajectories Among Chinese Adolescents
Source: Res Child Adolesc Psychopathol. 2021 May 13;49(10):1359–71. doi: 10.1007/s10802-021-00826-9 (PMC8380233; doi:10.1007/s10802-021-00826-9)
Supplement: Supplementary file 1 — Supplementary file1 (DOCX 20 KB) [file 10802_2021_826_MOESM1_ESM.docx]

**Appendix**

According to Cheung and Rensvold (2002), ΔCFI or ΔTFI >=.01 between consecutive models in invariance testing indicates a substantial deterioration in model fit. As shown in Table 1, the residual invariance model was supported because the changes in CFI and TLI were < .01, compared with the scalar model. Thus, consistent with previous findings (Stumper, Olino, Abramson, & Alloy, 2019), our results of longitudinal measurement invariance of the one-factor structure of CDI supported residual invariance (i.e., "strict invariance") across the four-time points. These findings indicate that changes in depressive symptoms during adolescence are due to true developmental changes, rather than changes in measurement.

Table 1 *Model fit indices for baseline models and measurement invariance models of CDI*

| One-factor Model | χ^2^ (*df)* | CFI | TLI | RMSEA | SRMR | ΔCFI | ΔTLI |
| --- | --- | --- | --- | --- | --- | --- | --- |
| T1, only | 1036 (324) | .98 | .98 | .03 | .04 | – | – |
| T2, only | 1208 (324) | .98 | .98 | .04 | .04 | – | – |
| T3, only | 1278 (324) | .97 | .97 | .04 | .04 | – | – |
| T4, only | 1208 (324) | .98 | .98 | .04 | .04 | – | – |
| Configural model | 12887 (4819) | .98 | .98 | .03 | .04 | – | – |
| Metric (weak invariance) | 14876 (4891) | .97 | .97 | .03 | .04 | < .01 | < .01 |
| Scalar (strong invariance) | 15335 (4963) | .98 | .97 | .03 | .04 | 0 | 0 |
| Residual (strict invariance) | 15554 (5038) | .97 | .97 | .03 | .04 | 0 | 0 |

Note: *χ*^2^= chi square; *df* = degrees of freedom; CFI = Comparative Fit Index; TLI = Tucker Lewis Index; RMSEA = Root Mean Square Error of Approximation; SRMR = Standardized Root Mean Square Residual. For all Models, *p*'s were < .001.

Reference:

Cheung, G. W., & Rensvold, R. B. (2002). Evaluating goodness-of-fit indexes for testing measurement invariance. *Structural Equation Modeling*, 9, 233-255. doi: 10.1207/s15328007sem0902_5

Stumper, A., Olino, T. M., Abramson, L. Y., & Alloy, L. B. (2019). A factor analysis and test of longitudinal measurement invariance of the children's depression inventory (CDI) across adolescence. *Journal of Psychopathology and Behavioral Assessment*, *41*, 692–698. https://doi.org/10.1007/s10862-019-09746-x
